# Supplementary material for: Clinico-Virological Outcomes and Mutational Profile of SARS-CoV-2 in Adults Treated with Ribavirin Aerosol for COVID-19 Pneumonia
Source: Microorganisms. 2024 Jun 5;12(6):1146. doi: 10.3390/microorganisms12061146 (PMC11205916; doi:10.3390/microorganisms12061146)
Supplement: Supplementary file 1 [file microorganisms-12-01146-s001.zip › microorganisms-2981307-supplementary.pptx]

## Slide 1
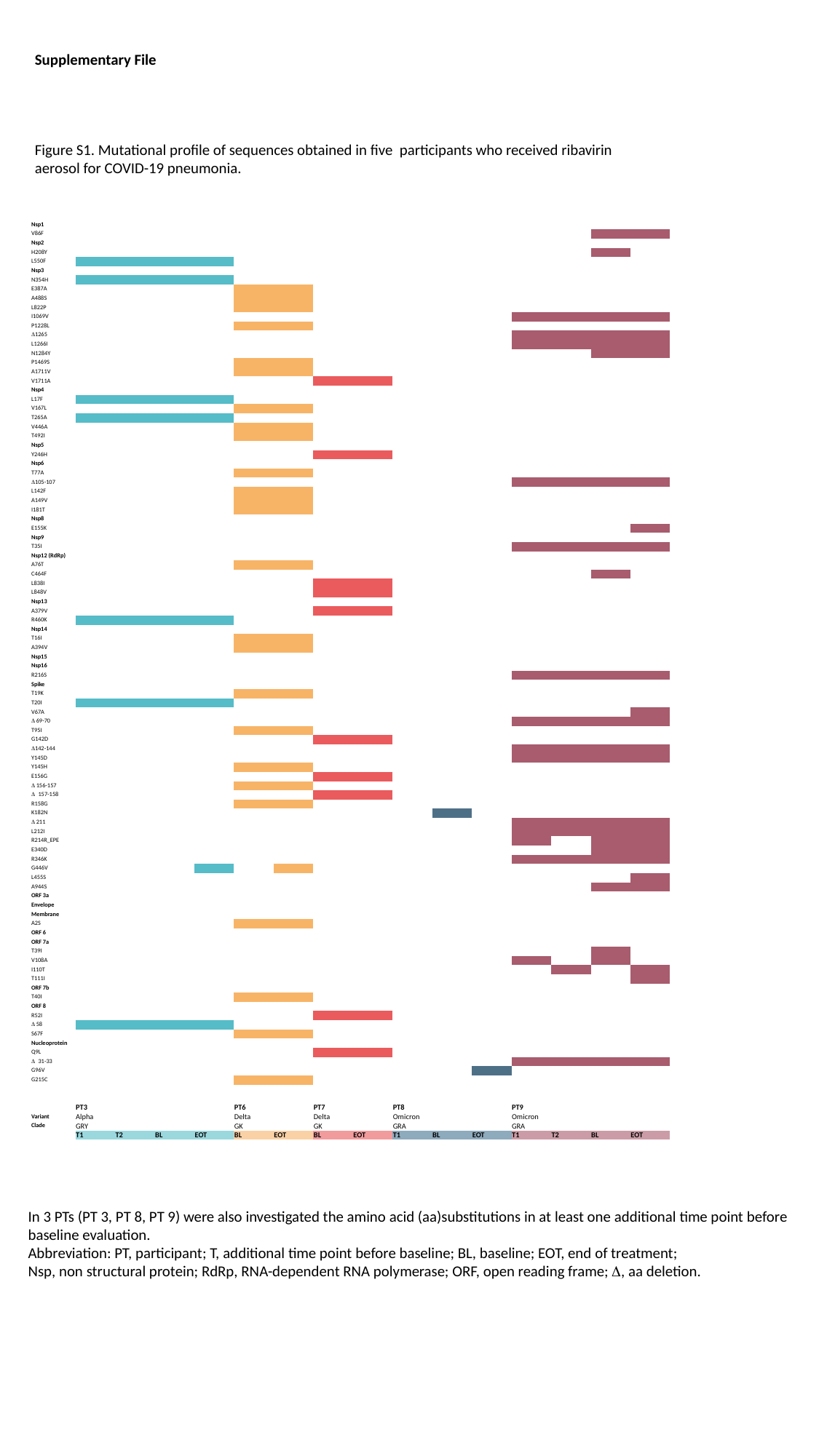

Supplementary File
Figure S1. Mutational profile of sequences obtained in five participants who received ribavirin aerosol for COVID-19 pneumonia.
| Nsp1 | | | | | | | | | | | | | | | |
| --- | --- | --- | --- | --- | --- | --- | --- | --- | --- | --- | --- | --- | --- | --- | --- |
| V86F | | | | | | | | | | | | | | | |
| Nsp2 | | | | | | | | | | | | | | | |
| H208Y | | | | | | | | | | | | | | | |
| L550F | | | | | | | | | | | | | | | |
| Nsp3 | | | | | | | | | | | | | | | |
| N354H | | | | | | | | | | | | | | | |
| E387A | | | | | | | | | | | | | | | |
| A488S | | | | | | | | | | | | | | | |
| L822P | | | | | | | | | | | | | | | |
| I1069V | | | | | | | | | | | | | | | |
| P1228L | | | | | | | | | | | | | | | |
| D1265 | | | | | | | | | | | | | | | |
| L1266I | | | | | | | | | | | | | | | |
| N1284Y | | | | | | | | | | | | | | | |
| P1469S | | | | | | | | | | | | | | | |
| A1711V | | | | | | | | | | | | | | | |
| V1711A | | | | | | | | | | | | | | | |
| Nsp4 | | | | | | | | | | | | | | | |
| L17F | | | | | | | | | | | | | | | |
| V167L | | | | | | | | | | | | | | | |
| T265A | | | | | | | | | | | | | | | |
| V446A | | | | | | | | | | | | | | | |
| T492I | | | | | | | | | | | | | | | |
| Nsp5 | | | | | | | | | | | | | | | |
| Y246H | | | | | | | | | | | | | | | |
| Nsp6 | | | | | | | | | | | | | | | |
| T77A | | | | | | | | | | | | | | | |
| D105-107 | | | | | | | | | | | | | | | |
| L142F | | | | | | | | | | | | | | | |
| A149V | | | | | | | | | | | | | | | |
| I181T | | | | | | | | | | | | | | | |
| Nsp8 | | | | | | | | | | | | | | | |
| E155K | | | | | | | | | | | | | | | |
| Nsp9 | | | | | | | | | | | | | | | |
| T35I | | | | | | | | | | | | | | | |
| Nsp12 (RdRp) | | | | | | | | | | | | | | | |
| A76T | | | | | | | | | | | | | | | |
| C464F | | | | | | | | | | | | | | | |
| L838I | | | | | | | | | | | | | | | |
| L848V | | | | | | | | | | | | | | | |
| Nsp13 | | | | | | | | | | | | | | | |
| A379V | | | | | | | | | | | | | | | |
| R460K | | | | | | | | | | | | | | | |
| Nsp14 | | | | | | | | | | | | | | | |
| T16I | | | | | | | | | | | | | | | |
| A394V | | | | | | | | | | | | | | | |
| Nsp15 | | | | | | | | | | | | | | | |
| Nsp16 | | | | | | | | | | | | | | | |
| R216S | | | | | | | | | | | | | | | |
| Spike | | | | | | | | | | | | | | | |
| T19K | | | | | | | | | | | | | | | |
| T20I | | | | | | | | | | | | | | | |
| V67A | | | | | | | | | | | | | | | |
| D 69-70 | | | | | | | | | | | | | | | |
| T95I | | | | | | | | | | | | | | | |
| G142D | | | | | | | | | | | | | | | |
| D142-144 | | | | | | | | | | | | | | | |
| Y145D | | | | | | | | | | | | | | | |
| Y145H | | | | | | | | | | | | | | | |
| E156G | | | | | | | | | | | | | | | |
| D 156-157 | | | | | | | | | | | | | | | |
| D 157-158 | | | | | | | | | | | | | | | |
| R158G | | | | | | | | | | | | | | | |
| K182N | | | | | | | | | | | | | | | |
| D 211 | | | | | | | | | | | | | | | |
| L212I | | | | | | | | | | | | | | | |
| R214R\_EPE | | | | | | | | | | | | | | | |
| E340D | | | | | | | | | | | | | | | |
| R346K | | | | | | | | | | | | | | | |
| G446V | | | | | | | | | | | | | | | |
| L455S | | | | | | | | | | | | | | | |
| A944S | | | | | | | | | | | | | | | |
| ORF 3a | | | | | | | | | | | | | | | |
| Envelope | | | | | | | | | | | | | | | |
| Membrane | | | | | | | | | | | | | | | |
| A2S | | | | | | | | | | | | | | | |
| ORF 6 | | | | | | | | | | | | | | | |
| ORF 7a | | | | | | | | | | | | | | | |
| T39I | | | | | | | | | | | | | | | |
| V108A | | | | | | | | | | | | | | | |
| I110T | | | | | | | | | | | | | | | |
| T111I | | | | | | | | | | | | | | | |
| ORF 7b | | | | | | | | | | | | | | | |
| T40I | | | | | | | | | | | | | | | |
| ORF 8 | | | | | | | | | | | | | | | |
| R52I | | | | | | | | | | | | | | | |
| D 58 | | | | | | | | | | | | | | | |
| S67F | | | | | | | | | | | | | | | |
| Nucleoprotein | | | | | | | | | | | | | | | |
| Q9L | | | | | | | | | | | | | | | |
| D 31-33 | | | | | | | | | | | | | | | |
| G96V | | | | | | | | | | | | | | | |
| G215C | | | | | | | | | | | | | | | |
| | | | | | | | | | | | | | | | |
| | | | | | | | | | | | | | | | |
| | PT3 | | | | PT6 | | PT7 | | PT8 | | | PT9 | | | |
| Variant | Alpha | | | | Delta | | Delta | | Omicron | | | Omicron | | | |
| Clade | GRY | | | | GK | | GK | | GRA | | | GRA | | | |
| | T1 | T2 | BL | EOT | BL | EOT | BL | EOT | T1 | BL | EOT | T1 | T2 | BL | EOT |
In 3 PTs (PT 3, PT 8, PT 9) were also investigated the amino acid (aa)substitutions in at least one additional time point before
baseline evaluation.
Abbreviation: PT, participant; T, additional time point before baseline; BL, baseline; EOT, end of treatment;
Nsp, non structural protein; RdRp, RNA-dependent RNA polymerase; ORF, open reading frame; D, aa deletion.
